# Comparative Evaluation of Microleakage of Bioactive, Ormocer, and Conventional GIC Restorative Materials in Primary Molars: An In Vitro Study Microleakage of Three Restorative Materials

**DOI:** 10.1155/2022/7932930

**Published:** 2022-03-11

**Authors:** Khushboo Jain, Farhin Katge, Manohar Poojari, Shilpa Shetty, Devendra Patil, Sanjana Ghadge

**Affiliations:** Department of Pediatric and Preventive Dentistry, TPCT's Terna Dental College, Navi Mumbai 400706, India

## Abstract

This in vitro study aimed to evaluate and compare the microleakage of bioactive, ormocer, and conventional glass ionomer cement (GIC) restorative materials in primary molars. In this study, class V cavities were prepared on the buccal surface of 75 noncarious extracted primary molars. The teeth were then restored as per the groups assigned. Group A, group B, and group C used bioactive restorative materials, ormocer restorative materials, and conventional GIC restorative materials for restorations, respectively. The teeth were then thermocycled and subjected to microleakage analysis via dye penetration. The microleakage scores were compared for differences using the Kruskal–Wallis test. This was followed by multiple pairwise comparisons using the Dunn test. All testing was carried out using a ‘p' value of <0.05. The percentage of samples showing microleakage score 0 depicting no dye penetration was highest for group A (56%) followed by group C (44%) and group B (12%). Statistical analysis revealed highest microleakage with group B, which was statistically significant (*p* < 0.05). Microleakage was evident in all the materials tested. The lowest microleakage was seen with bioactive restorative material.

## 1. Introduction

Recent advancements in adhesive dentistry have restricted the cavity size and shape to minimally invasive [1]. Despite the continuous evolution of restorative materials, problems such as marginal microleakage still persist. Microleakage may be defined as the clinically undetectable passage of bacteria, fluids, molecules, or ions between a cavity wall and the restorative material applied to it [2]. Adhesive restorative techniques are capable of bonding restorations micromechanically and biomechanically to the tooth surface, thereby reducing the chances of microleakage [3]. Conventional glass ionomer cement (GIC) is widely used for restoring posterior primary teeth due to its ease of use and acceptable esthetics [4]. GIC has undergone tremendous improvement with time. The cement has favorable properties such as chemical adhesion to dentin and enamel, fluoride release, coefficient of thermal expansion similar to dentin, and pulpal biocompatibility. However, the decreased wear resistance and low physical properties of GIC make their use limited in certain clinical conditions [5].

In the quest of finding a better restorative material, ormocers and bioactive materials were developed. Ormocers is an acronym for organically modified ceramics. The basic components of this material include silicones, organic polymers, and ceramic glasses [6]. A recent advance in the field of ormocers is the universal nanohybrid ORMOCER^®^ restorative material named Admira Fusion x-tra by Voco, Germany.

Initially, conventional restorative materials were thought to be biologically inert and biocompatible. Due to the emergence of bioactive materials in pediatric dentistry, they provide a promising alternative to conventional restorative materials. The interaction between bioactive materials and living tissue exhibits an elicit response, thus inducing the formation of hydroxyapatite [7]. One such bioactive dental material with an ionic resin matrix is ACTIVA^TM^ kids BioACTIVE restorative. It possesses a shock-absorbing resin component with bioactive glass fillers, similar to the properties of natural teeth. It is claimed to release more fluoride than glass ionomers [8, 9].

Microleakage remains a major cause of failure of restorations. It is the precursor of secondary caries, staining of restorations, tooth discoloration, marginal deterioration, postoperative sensitivity, and pulpal pathology [10].

Thus, this in vitro study aimed to evaluate and compare the microleakage of bioactive, ormocer, and conventional GIC restorative materials in primary molars. The null hypothesis proposed was that there would be no significant difference between the three materials in terms of microleakage.

## 2. Materials and Methods

This in vitro study was conducted during the period from June 2018 to December 2019 in the Department of Pediatric and Preventive Dentistry. The study was approved by the Institutional Review Board (IRB)–Ethics Committee. The sample size calculated per group was 25 primary molars with an alpha of 0.05 and a beta of less than 0.2 (power >80%). A total of 75 noncarious primary molars extracted for orthodontic intervention and teeth nearing exfoliation were selected for the study.

Each selected tooth underwent scaling and root planning with an ultrasonic device to remove residual organic tissue. Teeth were then stored in distilled water at room temperature until further use to prevent dehydration. Class V cavities were prepared on the buccal surface of the teeth one millimeter (mm) above the cementoenamel junction by an experienced operator. Cavity preparation was done using a high-speed airotor hand piece with a cylindrical diamond point. The cavity preparation was standardized on all teeth using a William's graduated periodontal probe. The dimensions of the cavity were three mm in length, two mm in width, and two mm in depth. The teeth were then divided randomly using a simple randomization technique into three groups based on the restorative material. Group A, group B, and group C used bioactive restorative material, ormocer restorative material, and conventional GIC restorative material for restorations, respectively. All restorations were performed according to the manufacturer's guidelines.

### 2.1. Procedure for Group a Restoration

Prepared cavities were acid etched for 10 seconds using Ultra-Etch 35% phosphoric acid gel (Ultradent Inc., USA) and then rinsed with water for 10 seconds. The cavities were then blotted with a cotton pellet to remove external moisture and avoid complete desiccation. Stae dentin/enamel single component total etch adhesive (SDI Limited, Australia), a fifth generation bonding agent was used. Adhesive was applied to the whole cavity followed by gentle air-drying to remove the excess. The adhesive was then light cured using an Ivoclar Vivadent Bluephase N *M* Light Cure Unit (New York, USA) for 20 seconds. The cavities were filled with bioactive restorative material named ACTIVA^TM^ kids BioACTIVE restorative material (Pulpdent® corporation, USA) using an ACTIVA-SPENSER dispensing gun (Pulpdent® corporation, USA). The restorations were light-cured for 20 seconds. The exposed restoration was covered with glycerin (an oxygen barrier) for it to self-cure. Finishing and polishing of restorations were then carried out using a composite polishing kit (Shofu Dental Corporation, USA).

### 2.2. Procedure for Group B Restoration

Prepared cavities were acid etched for 15 seconds using Ultra-Etch 35% phosphoric acid gel and then rinsed with water for 10 seconds 10. They were then air dried. A similar adhesive procedure was followed as per group A. The cavities were filled with an ormocer restorative material named Admira Fusion x-tra (Voco, Cuxhaven, Germany) and light cured using an Ivoclar Vivadent Bluephase N *M* Light Cure Unit (New York, USA) for 20 seconds. Finishing and polishing of restorations was carried out using a composite polishing kit (Shofu Dental Corporation, USA).

### 2.3. Procedure for Group C Restoration

Prepared cavities were conditioned for 20 seconds using 10% polyacrylic acid (Dentin Conditioner, GC Corporation, Tokyo, Japan) and then rinsed with water for 10 seconds. The cavities were then blotted with a cotton pellet to avoid complete desiccation. The cavities were filled with freshly mixed conventional GIC restorative material named GC Gold Label High Strength Posterior Restorative (GC Corporation, Tokyo, Japan). A coat of varnish (GC Fuji VARNISH, GC Corporation, Tokyo, Japan) was applied over the restorations once the initial set of material was achieved. Final finishing and polishing of restorations was carried out after 24 hours using a GIC finishing and polishing kit (Shofu Dental Corporation, USA). Again, a final coat of varnish was applied after the polishing of the restoration.

### 2.4. Thermocycling Procedure

The restored teeth were stored in distilled water at room temperature for one week. During this period, the teeth were subjected to 200 thermocycles between 5 °C and 55 °C in a water bath. Dwell time was 60 seconds with 10 seconds of transit between water baths [11, 12]. The samples were then prepared for microleakage evaluation of restorative materials.

### 2.5. Preparation of the Specimens for Microleakage Assessment

At the end of the test period, the apices of the teeth were sealed with sticky wax. All tooth surfaces except a one mm wide zone around the margins of the restoration were painted with two coats of nail varnish [11]. This was done for limiting dye penetration to the cavity margins. To minimize dehydration of the restorations, the teeth were placed in deionized water as soon as the nail varnish dried.

All teeth were then immersed in rhodamine B solution (1M) for 24 hours to allow dye penetration along the margins of restorative materials. The teeth were rinsed, dried, and invested in clear resin. Each tooth was sectioned buccolingually through the center of the restoration with the help of a low-speed water-cooled diamond disk. The specimens thus obtained were examined under 40X magnification in a stereomicroscope (SMZ-143 series, Motic Company Asia, Hong Kong) to evaluate the microleakage [12]. Dye penetration was graded based on the extent of penetration along the occlusal wall of the restoration. This was scored using criteria similar to the one used by Staninec and Holtz (1988) [13] .

### 2.6. Scoring Criteria [13] (Figure 1):

  Score 0—no dye penetration
  Score 1—dye penetration along the occlusal wall but less than halfway to the axial wall
  Score 2—dye penetration along the occlusal wall but more than halfway to the axial wall
  Score 3—dye penetration along the occlusal wall up to and along the axial wall

An independent investigator did the scoring of all samples to eliminate bias. The microleakage scores were compared for differences using the Kruskal–Wallis test, which is a nonparametric ANOVA. This was followed by multiple pairwise comparisons using the Dunn test. All testing was done using a ‘p' value of <0.05.

## 3. Results

**Figure 2:**
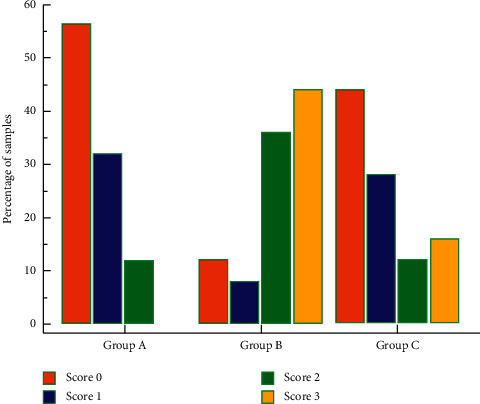
Colour bar graph depicting percentage of samples of all three groups showing particular microleakage score.

The results obtained from analyzing the data were as follows:

The total percentage of samples showing microleakage scores of 0, 1, 2, and 3 was 37.3%, 22.7%, 20%, and 20%, respectively. The percentage of samples showing microleakage score 0 was highest for group A (56%) followed by group C (44%) and group B (12%). Microleakage score 1 was seen in 32%, 8%, and 28% of samples of group A, group B, and group C, respectively. Microleakage scores 3 and 4 were seen more in samples of group B, 36% and 44%, respectively. The percentage of samples showing scores 3 and 4 in group A was 12% and 0%, respectively, and that in group C was 12% and 16%, respectively. It is shown graphically in Figure 2.

### 3.1. Intergroup Comparison

The microleakage scores were compared between the groups using the Kruskal–Wallis test. The results of the test are shown in Table 1. It showed that there is a significant difference (*p* = 0.000011) in the microleakage between bioactive, ormocer, and conventional GIC restorative materials in primary molars.

Microleakage scores were then compared between two groups at a time by multiple pairwise comparisons using the Dunn test. The lowest microleakage was seen with group A restoration. The result of this test showed that the microleakage of group B restoration was statistically different as compared to microleakage shown by group A (*p* < 0.0001) and group C (*p* = 0.0012) restoration. It was found that group A and group C restorations did not differ statistically (*p* = 0.1982) in their microleakage as shown in Table 2.

## 4. Discussion

Dental caries remains the most common dental disease, which affects children. The goal of restoring dental caries is to prepare and fill the cavity with an appropriate restorative material that restores form, function, and esthetics and prevents recurrence of caries. In pediatric dentistry, restorative materials that are fast setting and less technique sensitive with better properties are more preferred for restoring decayed primary teeth [14].

Most frequently encountered problem with restorations is the microleakage occurring at the tooth-restoration interface [15]. Many factors such as insufficient adhesion, polymerization shrinkage, inadequate moisture control, and incomplete removal of the smear layer, are responsible for the formation of marginal gaps between the cavity wall and the restoration [16]. Bioactive restorative material and ormocer restorative material are some of the recently developed materials with superior properties. At present, there is less literature that evaluates the microleakage of these newer materials in primary teeth.

The current in vitro study was carried out to evaluate and compare the microleakage of bioactive, ormocer, and conventional GIC restorative materials in primary molars. Extracted primary molars were used in this study. Primary teeth provide less bonding as compared to permanent teeth due to factors such as less inorganic content in the enamel, less intertubular dentin, and eventually have more microleakage [17, 18].

Class V restorations were chosen to evaluate microleakage in this study. More prismless enamel is present in the cervical region of primary teeth, which interferes with adequate bonding [19]. High C-factor, cyclic flexure, failure to bevel the enamel, composition, and structure of dentin are some of the factors which influence the likelihood of microleakage in class V cavities [20, 21].

A study by Longman et al. [22] and Bertrand et al. [23] showed no significant difference in microleakage between 200 cycles and 1000 cycles of thermocycling. Thus, in the current study, the restored teeth were subjected to 200 thermal cycles with a dwell time of 60 seconds at 5° and 55° with a transit time of 10 seconds.

The results of the present study showed some amount of microleakage in all three groups. These results were supported by Punathil S et al. They showed microleakage with the nanofilled resin-modified glass ionomer group, the nanocomposite resin group, and the Cention N group [24].

Bioactive materials show both light-activated and chemically activated polymerization, along with acid-base reaction [14]. ACTIVA^TM^ kids BioACTIVE restorative in the present study showed least microleakage when compared to other two restorative materials. The phosphate acid groups in the ionic resin component of ACTIVA^TM^ kids improve the interaction between the resin and the reactive glass fillers. This leads to an augmented interaction of ACTIVA^TM^ kids restorative to the tooth structure. Calcium ions from the hydroxyapatite crystals replaced the hydrogen ions which were removed from the phosphate groups through an ionization process. This ionic interaction between resin and the minerals of the tooth structure creates a protective seal, thus preventing microleakage [25].

A study by Omidi et al. in the year 2018 evaluated and compared the microleakage of Class II cavity restorations with ACTIVA^TM^ BioACTIVE restorative, resin-modified glass ionomer, and composite in primary molars. The study concluded that microleakage of ACTIVA^TM^ BioACTIVE restorative material in the absence or presence of etching and bonding could be comparable to the microleakage of composites [26].

Application of a bonding agent is only recommended for nonretentive cavities by manufacturers. Thus, in the present study, due to the nonretentive nature of class V cavities, Stae dentin/enamel single component total etch adhesive (SDI Limited, Australia), a fifth generation bonding agent was used. A study by Kaushik and Yadav [25] in the year 2017 compared and evaluated microleakage in class V lesions restored with ACTIVA^TM^ BioACTIVE restorative and nanohybrid composite resin using two different bonding agents (tetric N bond and *G* bond). The authors concluded that ACTIVA^TM^ BioACTIVE restorative when used in combination with tetric N bond showed the least microleakage. Tetric N bond is also a fifth generation bonding agent [25]. Hence, in the present study, the use of fifth generation bonding agents might have further contributed to low microleakage scores. However, a study by Cannavo et al. suggested that ACTIVA^TM^ BioACTIVE restorative applied without a bonding agent “compared favorably” to conventional composite resins placed with bonding agents [27].

A study by Alkhudhairy and Ahmad [1] on Class II cavities of maxillary premolars depicted a higher microleakage in ACTIVA^TM^ BioACTIVE Restorative than in SureFil SDR® composite (Dentsply, USA). The difference between the findings of their study and the present study may be attributed to the different types of teeth and restorative materials used [1]. A randomized controlled trial by Bhadra et al. [28] in the year 2018 evaluated and compared the clinical performance of a nanohybrid composite with ACTIVA^TM^ BioACTIVE restorative material in Class II cavities of permanent molars. The trial concluded that both materials showed equal and acceptable clinical performance at the end of 1 year [28].

Ormocer is a new type of hybrid dental composite composed of organic, inorganic, and polysiloxane parts [7]. This material shows the lowest level of polymerization shrinkage (1.25% by volume) and extremely low shrinkage stress when compared to conventional composite resins [29]. In vivo study by AI-Harbi et al. compared the microleakage values of an ormocer based material and a commonly used composite resin. The authors found no significant difference in the degree of microleakage between the two materials [30]. Ormocer, nanohybrid, nanofill resin composite, and microhybrid composite showed equally acceptable clinical performance in restorations of small occlusal cavities of posterior teeth after two years [31]. In a study with Class II cavities, ormocer showed less polymerization shrinkage than hybrid composites [32]. Sudhapalli SK et al. in their in vitro study concluded that the marginal sealing ability of ormocer was the least as compared to composite resins in class V cavities [33]. Kalra et al. compared ormocer (Admira) and hybrid composite marginal sealing ability using two types of bonding agents. The two bonding agents used in their study were ormocer-based bonding agent and conventional fifth generation bonding agents. Admira with an ormocer-based bonding agent exhibited the least microleakage as compared to other groups [31]. Similarly, Shathi et al. showed less microleakage with ormocer when compared to giomer [34].

However, in the present study, the highest microleakage was seen with ormocer restorative material. This might be attributed to multiple factors such as inadequate adaptation of the restorative material with tooth tissue, polymerization shrinkage, and air entrapment during placement of the restorative material. Also, few studies have been conducted with ormocer on primary teeth; hence, more research is needed. In the present study, the conventional GIC restorative material showed moderate levels of microleakage. This may be attributed to poor physical properties to withstand thermal stresses. Also, conventional GIC is the most moisture-sensitive restorative material in the early stages of placement [35]. Ayna B et al. examined the microleakage of dye at the edges of primary tooth restorations using three glass ionomer-based restorative materials. They found that conventional GIC exhibited the highest microleakage as compared to the other two variants of GIC which included resin-modified glass ionomer cement and polyacid-modified composite resin [36].

## 5. Limitation

Direct correlation between the results of dye penetration studies and the clinical outcome appears to be difficult. Therefore, in vitro studies cannot replace clinical studies, but can serve as a guideline to predict microleakage of a material in a clinical situation. Microleakage is one of the most important factors, which can determine the success of restorative dentistry. In the current study, variations in the microleakage were observed. Factors such as bonding ability of the material to the tooth surface and solubility of the material in the oral fluids play a major role in determining the success of the restoration. Thus, the quest for an ideal restorative material is an ongoing process. In addition, more research is required for estimating the degree of microleakage in an oral environment.

## 6. Conclusion

Based on the findings of the present study, the following conclusions can be made:

1. All restorative materials tested in this in vitro study exhibited some amount of microleakage in primary molars
2. Ormocer restorative material showed the highest microleakage followed by conventional GIC and bioactive restorative material in primary molars
3. Bioactive restorative material can be used as an effective alternative restorative material for primary molars

More research is needed in this aspect.

## Figures and Tables

**Figure 1:**
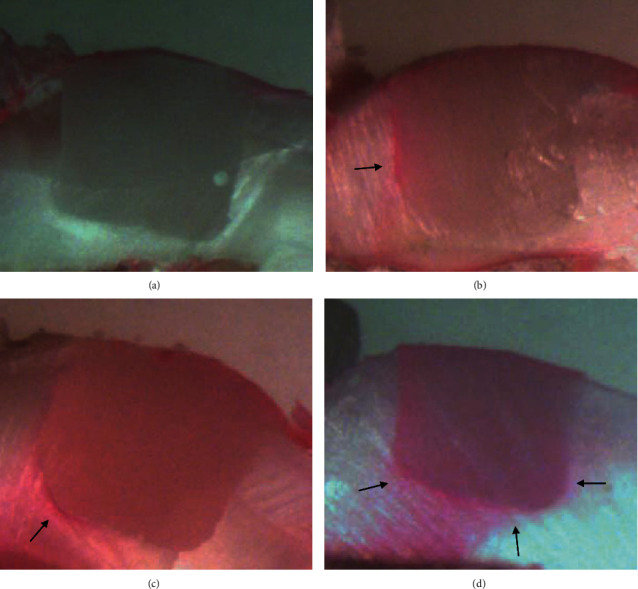
Dye penetration scores under 40x magnification. (a) Score 0 (b) Score 1. (c) Score 2. (d) Score 3.

**Table 1:** Kruskal–Wallis test.

Test statistic	21.0038
Degrees of freedom (DF)	2
Significance level	*p* = 0.000011

**Table 2:** Post hoc analysis using the Dunn test.

Factor	n	Average rank	Different (*p* < 0.05) from factor nr
(1) Group A	25	26.32	(2)
(2) Group B	25	53.70	(1) (3)
(3) Group C	25	33.98	(2)

## Data Availability

The data supporting the findings of the study are available from the corresponding author upon reasonable request.
